# Exploring a potential palonosetron allosteric binding site in the 5-HT_3_ receptor^☆^

**DOI:** 10.1016/j.bmc.2013.09.028

**Published:** 2013-12-01

**Authors:** Marta Del Cadia, Francesca De Rienzo, David A. Weston, Andrew J. Thompson, Maria Cristina Menziani, Sarah C.R. Lummis

**Affiliations:** a Dipartimento di Scienze Chimiche e Geologiche, Università degli Studi di Modena e Reggio Emilia, Via Campi 183, 41100 Modena, Italy; b Department of Biochemistry, University of Cambridge, Tennis Court Road, Cambridge CB2 1QW, UK

**Keywords:** 5-HT, 5-hydroxytryptamine (serotonin), EC_50_, concentration of agonist required for half-maximal response, HEK, human embryonic kidney, IC_50_, concentration of antagonist required for half-maximal inhibition, *K*_d_, affinity constant, Serotonin receptor, Allosteric binding site, Site-directed mutagenesis, Radioligand binding, FlexStation assays, Palonosetron, Computational studies

## Abstract

Palonosetron (Aloxi) is a potent second generation 5-HT_3_ receptor antagonist whose mechanism of action is not yet fully understood. Palonosetron acts at the 5-HT_3_ receptor binding site but recent computational studies indicated other possible sites of action in the extracellular domain. To test this hypothesis we mutated a series of residues in the 5-HT3A receptor subunit (Tyr^73^, Phe^130^, Ser^163^, and Asp^165^) and in the 5-HT3B receptor subunit (His^73^, Phe^130^, Glu^170^, and Tyr^143^) that were previously predicted by in silico docking studies to interact with palonosetron. Homomeric (5-HT_3_A) and heteromeric (5-HT_3_AB) receptors were then expressed in HEK293 cells to determine the potency of palonosetron using both fluorimetric and radioligand methods to test function and ligand binding, respectively. The data show that the substitutions have little or no effect on palonosetron inhibition of 5-HT-evoked responses or binding. In contrast, substitutions in the orthosteric binding site abolish palonosetron binding. Overall, the data support a binding site for palonosetron at the classic orthosteric binding pocket between two 5-HT_3_A receptor subunits but not at allosteric sites previously identified by in silico modelling and docking.

## Graphical abstract

**Figure:**
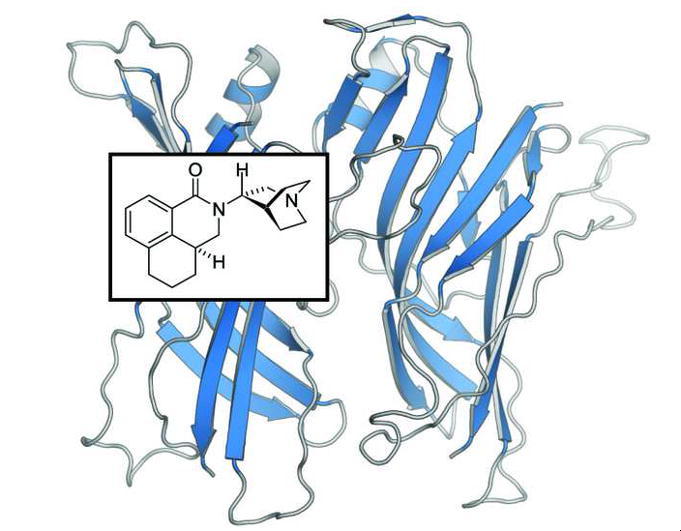

## 1 Introduction

Cys-loop receptors constitute one of the largest families of ligand-gated ion channels responsible for the transmission of fast synaptic impulses in the CNS and PNS. The nicotinic acetylcholine, glycine, GABA_A_ and 5-HT_3_ receptors, all belong to this family, sharing homologous amino acid sequences and similar arrangements of subunits.^1,2^ The 5-HT_3_ receptor is formed by a pentameric assembly of identical (homomeric) or different (heteromeric) subunits surrounding a central ion-conducting pore. Each subunit has an extracellular domain, a transmembrane domain and an intracellular domain. The binding pocket is formed between two adjacent subunits, and is constituted of the + (or principle) face of one subunit and the − (or complementary) face of the adjacent subunit (e.g., A+A− when between two 5-HT3A receptor subunits). To date, five different 5-HT_3_ receptor subunits have been identified. Receptors that contain only 5-HT3A receptor subunits (5-HT_3_A receptors) or 5-HT3A and 5-HT3B receptor subunits (5-HT_3_AB receptors) have been widely investigated, but receptors containing 5-HT3C, 5-HT3D and 5-HT3E receptor subunits have been less well characterized.^1–8^

Radioligand binding and in situ hybridization studies have shown that 5-HT_3_ receptors are present throughout the nervous system, and play a role in a variety of nervous and sensory functions, as well as in emesis, cognition and anxiety.^1,3,4^ A number of 5-HT_3_ receptor antagonists are therapeutic agents, including ondansetron, tropisetron, granisetron and palonosetron, with major uses in the management of chemotherapy-induced, radiation-induced and post-operative nausea and vomiting.^5^

Palonosetron is the newest 5-HT_3_ receptor antagonist licensed for clinical use and demonstrates improved control of acute (0–24 h) and delayed (24–120 h) emesis after moderate chemotherapy administration when compared to early generation antagonists such as granisetron (Kytril). Palonosetron also has a higher in vitro binding affinity for the 5-HT_3_ receptor compared to other antagonists (p*K*_i_ of 10.5 vs 8–9), and an extended in vivo plasma elimination half-life.^6,7^ Palonosetron has unique structural characteristics when compared to other drugs in its class and data suggest that palonosetron interacts with 5-HT_3_ receptors differently to granisetron or ondansetron, with early studies indicating allosteric effects and positive cooperativity.^12,13^ These authors also provided data that suggests palonosetron triggers receptor internalisation, resulting in a long-lived inhibition of receptor function, although more recent data show that the dissociation of palonosetron is very slow, which could explain this effect.^8,9^

**Figure 1:**
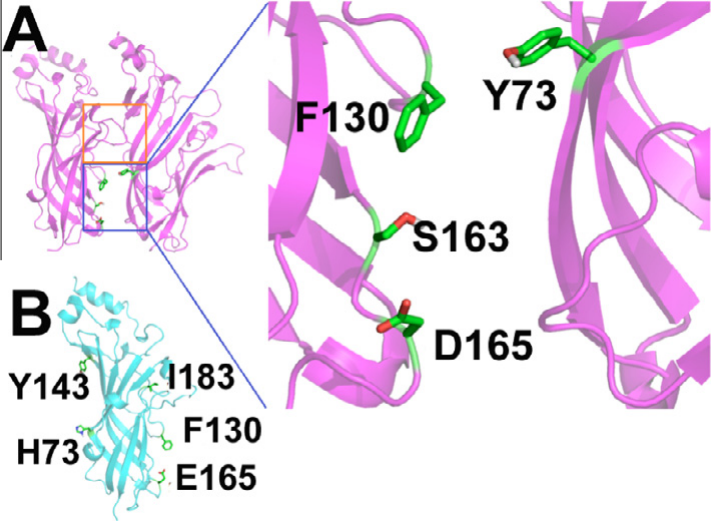
(A) A homology model of the human 5-HT_3_A receptor showing the 5-HT binding site (orange square), a potential allosteric binding site (blue square) and the location of the 5-HT_3_A subunit residues (stick representation) mutated in this study; inset: the potential allosteric binding site predicted by homology modelling and in silico docking, showing the 5-HT_3_A subunit residues mutated in this study; (B) homology model of the human 5-HT_3_B subunit showing the residues mutated in this study.

**Figure 2:**
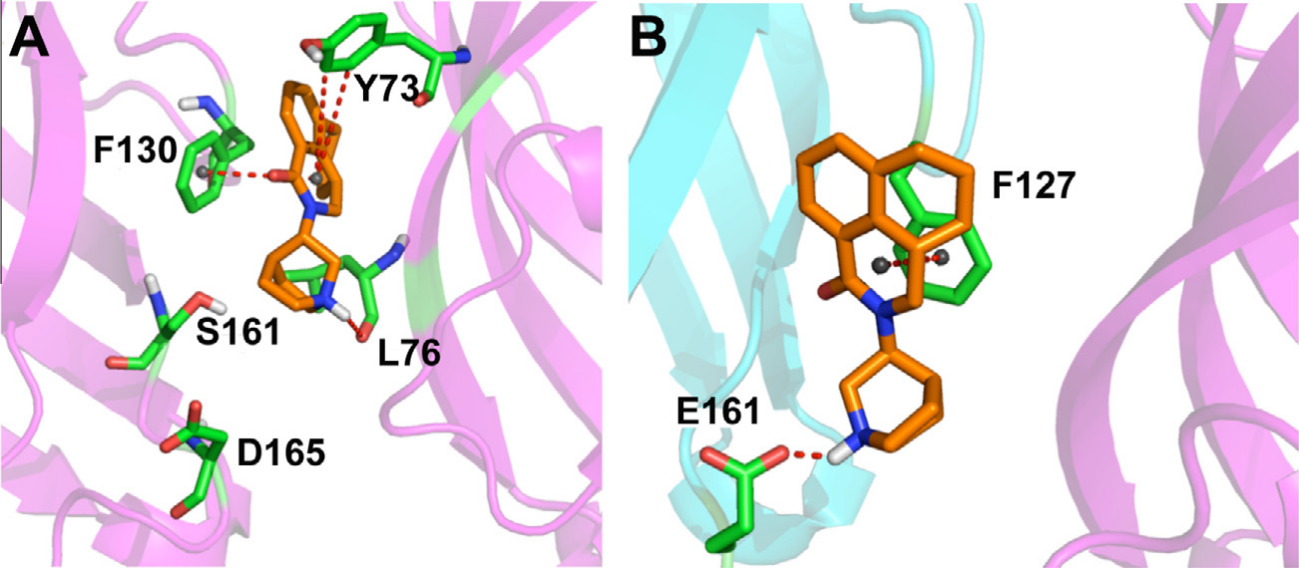
Previous predicted binding mode of palonosetron into the A+A− (A), and B+A− (B) interfaces, showing the interactions identified these in silico studies.^10^

Recent computational work modelled the extracellular domain of the human 5-HT_3_A and 5-HT_3_B subunits assembled into homo- and heteromeric receptors to yield A+A− and B+A− interfaces.^10^ Docking of serotonin, palonosetron and granisetron into these binding interfaces was used to probe the binding characteristics of palonosetron (Fig. 1), with the aim of providing a viable explanation for the higher efficacy of palonosetron, as well as investigating the allosteric binding and positive cooperativity. These studies suggested a second binding site for palonosetron at the A+A− interface that was not found for granisetron; this was located below the 5-HT binding site, closer to the membrane. Palonosetron was also proposed to bind to a B+A− interface. In silico studies such as these are increasingly being used to probe the mechanism of action of drugs, but because many of these use homology models, it is essential such data are confirmed using experimental studies. The present work uses HEK293 expressed mutant 5-HT_3_A and 5-HT_3_AB receptors to experimentally probe the residues identified as interacting with palonosetron in the previously published in silico study (Fig. 2, Table 1).

## 2 Materials and methods

### 2.1 Materials

All cell culture reagents were obtained from Gibco BRL (Paisley, UK), except foetal calf serum which was from Labtech International (Ringmer, UK). [^3^H]Granisetron (84.5 Ci/mmol) was from PerkinElmer (Boston, MA). FlexStation membrane potential dye was from Molecular Devices Ltd (Wokingham, UK). [^3^H]Palonosetron (37.2 Ci/mmol) was custom synthesised for Helsinn Healthcare (Lugano, Switzerland), and both this and the unlabelled form of palonosetron were kindly gifted by Helsinn Healthcare (Lugano, Switzerland). All other reagents were of the highest obtainable grade.

### 2.2 Cell culture

Human embryonic kidney (HEK) 293 cells were cultured on 90 mm tissue culture plates at 37 °C and 7% CO_2_ in a humidified atmosphere. They were grown in DMEM:F12 (Dulbecco’s Modified Eagle’s Medium/Nutrient Mix F12 (1:1)) with GlutaMAX I™ containing 10% fetal calf serum and passaged when confluent. For radioligand binding studies, cells in 90 mm dishes were transfected at 60–70% confluency using polyethyleneimine and incubated for 3–4 days before use. For FlexStation assays, transfected cells were plated on 96-well plates and incubated 1–2 days before assay.

### 2.3 Site-directed mutagenesis

Mutagenesis reactions were performed using either the Kunkel method or the QuikChange kit (Agilent Technologies Inc., CA, USA) using human 5-HT3A or 5-HT3B receptor subunit cDNA (accession numbers: P46098 and O95264) in pcDNA3.1 (Invitrogen, Paisley, UK). Subunit numberings have been altered to the aligning residues in the mouse 5-HT3A subunit for ease of comparison with other studies.

### 2.4 Radioligand binding

Transiently transfected cells were washed twice with 2 ml phosphate-buffered saline (PBS), harvested into 1 ml cold HEPES buffer (10 nM, pH 7.4) containing protease inhibitors (1 mM EDTA, 50 μg/ml soybean trypsin inhibitor, 50 μg/ml bacitracin and 0.1 mM phenylmethylsulphonyl fluoride) and frozen at −20 °C. Once thawed, membranes were washed and resuspended in ice-cold HEPES buffer.

Eight-point binding assays were performed to produce saturation binding curves. Membrane suspensions (50 μl) in 0.5 ml HEPES buffer were incubated at 4 °C with 0.05–2 nM [^3^H]-granisetron (for 1 h) or [^3^H]-palonosetron (for 24 h). Unlabelled quipazine (0.1 mM) was used to determine non-specific binding. Membranes were harvested onto presoaked (0.3% polyethyleneimine) GF/B filters using a Brandel harvester, followed by two 2 ml washes of ice-cold HEPES buffer. Radioactive counts were measured using a Beckman LS6000SC liquid scintillation counter (Fullerton, California, USA).

Data were analyzed by iterative curve fitting using GraphPad Prism v5 (San Diego, CA) according to the following equation:

$$B=\frac{B_{\max}{\left[L\right]}^{n}}{k_{\text{d}}^{n}+{\left[L\right]}^{n}}$$

where *B* is the amount of bound radioligand, *B*_max_ is the maximum specific binding at equilibrium, [*L*] is the concentration of radioligand, *K*_d_ is the equilibrium dissociation constant of the radioligand, and n is the Hill coefficient.

### 2.5 Functional studies

Fluorometric studies were undertaken using a FlexStation® (Molecular Devices, Sunnyvale, CA, USA) as previously described.^11^ Transfected cells grown on 96-well plates were washed using a standard buffer (10 mM HEPES, 115 mM NaCl, 1 mM KCl, 1 mM CaCl_2_, 1 mM MgCl_2_, 10 mM glucose, pH 7.4), loaded with FLIPR® Membrane Potential Dye (Molecular Devices) according to the manufacturer’s instructions, and then incubated at 37 °C for 45 min before being assayed.

Fluorescence was measured every 2 s for 200 s. At 20 s, 50 μl of buffer or 5-HT (final concentration 0.003 μM to 1.0 mM) was added to each well. For experiments using palonosetron (0.01 nM–3.0 μM) the antagonist was incubated for 45 min with the cells before starting the experiment using an EC_50_ concentration of 5-HT (unless otherwise stated). Concentration–response and inhibition curves were obtained for each mutant, and the fluorescence values were normalized to the maximal response. The data were fitted using GraphPad Prism v5.0 using the equation:

$$F=F_{\min}+\frac{\left(F_{\max}-F_{\min}\right)}{1+10^{\left(\log({\text{EC}}_{50}-[L])n_{\text{H}}\right)}}$$

where *F* is the fluorescence, *F*_max_ is the maximum response, *F*_min_ is the baseline fluorescence, [*L*] is the ligand concentration, *n*_H_ is the Hill coefficient, and the EC_50_ is the ligand concentration required to obtain 50% of maximal fluorescence.

### 2.6 Statistical analysis

Values are presented as mean ± SEM. Statistical analysis was performed using ANOVA in conjunction with a Dunnett’s post-hoc test; *p* <0.05 was taken as statistically significant.

## 3 Results

### 3.1 Probing the novel palonosetron binding site

#### 3.1.1 [^3^H]Granisetron radioligand binding

##### 3.1.1.1 Wild type receptors

[^3^H]-granisetron saturation binding experiments showed similar binding affinity for homo- and heteromeric receptors, (*K*_d_ = 0.53 ± 0.15 nM and 0.20 ± 0.03 nM, respectively) consistent with data previously reported.^12,13^

##### 3.1.1.2 Mutant homomeric receptors

Mutant receptors (Y73A, Y73F, Y73S, F130A, F130Y, S163A, S163T, D165A and D165K) had *K*_d_ values were not significantly different to wild type (Table 2).

##### 3.1.1.3 Mutant heteromeric receptors

Most receptors containing mutations in the 5-HT_3_B receptor subunit (H73A, Y143A, and E170A) had *K*_d_ values that were not significantly different to wild type. F130A containing heteromers showed a small (∼fourfold) increase in *K*_d_ compared to wild type receptors (Table 2).

#### 3.1.2 [^3^H]Palonosetron radioligand binding

##### 3.1.2.1 Wild type receptors

[^3^H]Palonosetron bound with similar high affinity to 5-HT_3_A and 5-HT_3_AB receptors (*K*_d_ = 0.32 ± 0.07 and 0.18 ± 0.07 nM, respectively) (Table 2), a finding consistent with previous studies.^6,7^

##### 3.1.2.2 Mutant homomeric receptors

Most mutant receptors (Y73F, F130A, F130Y, S163A, S163T, D165A and D165K) had *K*_d_ values similar to wild type. There was a small increase in *K*_d_ (∼2.5-fold) for Y73A receptors.

##### 3.1.2.3 Mutant heteromeric receptors

Most mutant receptors containing subunits with 5-HT_3_B subunit mutations (H73A, W90C, Y143A, and E170A) had *K*_d_ values similar to wild type, but there was a small increase (∼twofold) in *K*_d_ for F130A containing heteromers.

#### 3.1.3 Functional assays

##### 3.1.3.1 Wild type receptors

Our functional responses of WT 5-HT_3_AB receptors expressed in HEK cells and studied using membrane potential sensitive dye show a similar EC_50_ and a decrease in Hill slope (EC_50_ = 0.5 μM, *n*_H_ = 1.3) when compared to wild type 5-HT_3_A receptors (EC_50_ = 0.32 μM, n_H_ = 2.3). These data are similar to previous reports.^14–16^

##### 3.1.3.2 Mutant homomeric receptors

One of the mutant receptors, F130Y, was non-functional. As this mutant had binding characteristics similar to wild type, this indicates that the presence of Y at this position produces non-functional receptors. Y73A and Y73S mutant receptors had lower EC_50_ values than wild type receptors. All other mutant receptors had EC_50_ values that were not significantly different to wild type (Table 3).

Inhibition studies with palonosetron revealed its IC_50_ was increased (∼15-fold) at Y73A containing mutant receptors compared to wild type receptors. No other receptors were different to wild type receptors (Table 4).

##### 3.1.3.3 Mutant heteromeric receptors

None of the heteromeric receptors had 5-HT EC_50_ values significantly different to wild type receptors, and none had differences in IC_50_ values for palonosetron (Tables 3 and 4).

### 3.2 Probing the orthosteric binding site

To probe the orthosteric binding site we examined [^3^H]palonosetron binding to receptors that had substitutions at two critical binding site residues in the 5-HT3A subunit; W183 in loop B, and W90 in loop D. The binding characteristics of receptors containing Cys substitutions at these locations revealed no saturable, specific binding with [^3^H]palonosetron (Table 2). This is consistent with previously published data from [^3^H]granisetron binding studies, and indicates that palonosetron binds in this pocket. Lack of specific [^3^H]palonosetron binding could alternatively indicate that the mutant receptors were not expressed, but we consider this is unlikely as a previous work has demonstrated cell surface expression of 5-HT_3_ receptors with mutations of W90 and W183.^17^

To probe possible binding to A+B−, A+B−, B+A− or B+B− interfaces in 5-HT_3_AB receptors, we also substituted the equivalent residues in the 5-HT_3_B subunit (I183 and W90) and co-expressed them with wild-type 5-HT3A subunits. [^3^H]palonosetron binding data revealed that binding affinities and functional responses were unaltered in these modified 5-HT_3_AB receptors.

## 4 Discussion

Palonosetron is a potent 5-HT_3_ receptor antagonist that has improved properties for ameliorating the symptoms of chemotherapy-induced and post-operative nausea and vomiting when compared to earlier antagonists. A possible explanation for this high potency is the presence of an allosteric binding site, and such a site was suggested in a computational study by Moura Barbosa et al.^10^ Here we used mutagenesis to probe the proposed interactions between palonosetron and residues in the second A+A− binding site and those in the 5-HT_3_B containing binding sites; the data confirm previous studies that show palonosetron binds to the orthosteric binding site located between two adjacent 5-HT_3_A subunits (with no contribution from 5-HT_3_B subunits), but do not support an alternative binding location.^6,7,18,13^ There was some indication that Y73 had an interaction with palonosetron, although the effects of mutating this residue were small. The roles of each of the residues that we examined are discussed in more detail below.

Y73 is located just below the binding pocket and in the model presented by Moura Barbosa et al.,^10^ shows a side to edge interaction with palonosetron (Fig. 2). Our substitutions here caused a small increase in the *K*_d_ for palonosetron binding and the IC_50_ for inhibition of 5-HT-induced responses, suggesting that Y73 does not have a major interaction with palonosetron, but may influence the orthosteric site above, or may form part of a temporary binding location on route to the ‘classic’ binding pocket; a simulation study showing the trajectory of granisetron as it unbinds from the receptor indicates that ligands exits below the binding site close to Y73,^19^ although altering the equivalent residue in mouse 5-HT_3_ receptors has no effect on [^3^H]granisetron binding.^20^ Therefore, it is more likely that this residue is structurally important, as suggested in a recent computational work, where molecular dynamics simulations and computational alanine scanning mutagenesis revealed that Y73 is part of the ‘hot centre’ of the subunit–subunit interaction, an aromatic cluster located in the middle of the binding interface involved in the stabilization of the protein.^21,22^ Further evidence for a structural role for this residue comes from our EC_50_ values, which are lower for Y73A and Y73S mutant receptors.

F130 is located on loop A and has been extensively investigated in mouse 5-HT_3_A receptors, where it has been shown to have an important role in function: mutation to Ala or Trp decreases the 5-HT EC_50_ yet increases the granisetron *K*_d_, while mutation to Tyr increases the EC_50_ but has no effect on the *K*_d_.^23,24^ These data suggest that this residue does not directly bind ligand, but that adding a hydroxyl is deleterious to receptor function; our results from human 5-HT_3_ receptors reveal a similar but more pronounced effect as F130Y-containing receptors are expressed but are non-functional. The equivalent residue in the 5-HT_3_B subunit was the only mutated residue (F130A) that caused an increase in granisetron binding affinity. There is currently no evidence that granisetron binds to a 5-HT_3_B subunit-containing binding pocket, and we suggest that this mutation causes a subtle change in receptor structure that influences the ligand binding (A+A−) interface. Support for this hypothesis comes from our palonosetron binding data, which reveals a small increase in affinity in receptors containing F130A-containing 5-HT_3_B receptor subunits. There was, however, no change in EC_50_ suggesting no significant effect on agonist binding and/or channel gating. There was also no effect on palonosetron inhibition of function, suggesting the inhibitory effect of palonosetron does not involve binding to F130.

S163 is located below the binding pocket and was predicted to be <5 Å from palonosetron in the allosteric site proposed by Moura Barbosa et al.,^10^ but not directly involved in its binding. Our data showed that mutation of S163 to either Ala or Thr resulted in no significant changes to binding or functional parameters, suggesting this residue does not play a significant role in palonosetron binding or receptor function. This is in agreement with a previous study that also concluded that there was no involvement of this Ser in channel activation or in coupling of ligand binding to channel gating as this residue was found to be a ‘warm spot’ only involved in subunit–subunit interactions stabilising the receptor.^21^

D165 lies further from the orthosteric binding pocket than the residues discussed above but is a potentially novel palonosetron binding site identified in the Moura Barbosa et al. study,^10^ and is also on the unbinding pathway for 5-HT and granisetron.^19^ However our data revealed that mutation of D165 to either Ala or Lys resulted in no significant changes to binding or functional parameters, suggesting this residue does not play a role in palonosetron binding.

The docking study of Moura Barbosa et al.^10^ was aimed at probing whether ligands could bind to an A+A− or a B+A− interface. These interfaces were chosen because the critical residues that differ between 5-HT_3_A and 5-HT_3_B receptor subunits are on the principal face, and thus A+A− would be equivalent to A+B−, and B+A− equivalent to B+B−, covering all possible subunit interface combinations that could occur in a 5-HT_3_AB receptor; furthermore computational studies have examined the role played by the extracellular moieties of the A and B subunits in the formation of functional or non functional receptors, which have been analyzed in terms of the hydrophobic and electrostatic properties of the different subunits and their complementarity.^25^ At that time the stoichiometry of the receptor had been proposed to be BBABA,^25^ but more recent data has shown that functional receptors require an A+A− interface and the heteromeric stoichiometry is 3A2B, with a likely subunit arrangement of AABAB^26,13,27^ Our current data show the 5-HT_3_B subunit mutations have little or no effect on receptor binding or function, consistent with palonosetron binding only to an A+A− interface, although there may be some allosteric effects of 5-HT_3_B subunit mutations, as we have proposed for F130. A 5-HT_3_ antagonist that does interact with both the A+A− and A+B− interfaces has recently been identified.^28^ This compound (VUF10166) shows modified binding and functional effects when tested on A+B− receptor containing mutant 5-HT_3_B subunits, indicating an allosteric effect at an A+B− interface, although it also competed for granisetron at the A+A- binding site.

## 5 Conclusions

In conclusion our data support a binding location for palonosetron in the orthosteric (A+A−) binding site in both homomeric 5-HT_3_A and heteromeric 5-HT_3_AB receptors, but do not provide evidence for a second binding site for palonosetron at either the proposed allosteric site at the A+A− binding site or at the B+A− interface. The question remains as to whether there is another palonosetron binding site elsewhere in 5-HT_3_A or 5-HT_3_AB receptors. This is potentially possible but recent data suggest that at least some of the unusual effects of palonosetron can be explained by its unusual kinetics, and thus we consider that a second binding site is unlikely.

## Figures and Tables

**Table 1:** Human 5-HT_3_R residues mutated in this study and their corresponding residues in mouse

Mutated residue (mouse numbering)	Human numbering	ECD Loop	Predicted docking interaction	Mutant
*5-HT_3_A subunit mutations*				
Y73	Y68		Hydrophobic/aromatic interactions.	A F S
W90	W85	D	Aromatic box component	C
F130	F125	A	π–π interaction with the aromatic region	A Y
S163	S158	Cys	<5 Å	A T
D165	D160	Cys	Possibility of a charge-reinforced H-bond with the cationic head	A K
W183	W178	B	Aromatic box component	C

*5-HT_3_B subunit mutations*				
H73	H66			A
W90	W83	B	Aromatic box component	C
F130	F123	A	π–π interaction with the aromatic region	A
Y143	Y136	E		A
E165	E158	Cys	Charge-induced H bond with the primary amine	A
I183	I176	B	Aromatic box component	A

**Table 2:** Affinities of mutant 5-HT_3_A and 5-HT_3_AB receptors for [^3^H]-granisetron and [^3^H]palonosetron

Mutant	Granisetron		Palonosetron	
	*K*_d_ (nM)	*n*	*K*_d_ (nM)	*n*
*5-HT_3_A subunit mutations*				
WT 5-HT_3_A	0.53 ± 0.15	5	0.32 ± 0.07	4
Y73A	0.60 ± 0.11	4	0.87 ± 0.04⁎	3
Y73F	0.90 ± 0.29	4	0.25 ± 0.04	3
Y73S	0.41 ± 0.11	4	0.61± 0.11	5
W90C	NB	8	NB	3
F130A	0.78 ± 0.26	3	0.67 ± 0.12	4
F130Y	0.49 ± 0.17	4	0.26 ± 0.09	3
S163A	0.36 ± 0.19	3	0.30 ± 0.08	4
S163T	0.19 ± 0.09	6	0.22 ± 0.05	6
D165A	0.37 ± 0.11	4	0.30 ± 0.08	3
D165 K	0.44 ± 0.09	5	0.36 ± 0.02	4
W183C	NB	6	NB	3

*5-HT_3_B subunit mutations*				
WT 5-HT_3_AB	0.20 ± 0.03	4	0.18 ± 0.02	4
H73A	0.22 ± 0.02	3	0.14 ± 0.02	3
W90C	0.25 ± 0.10	4	0.34 ± 0.06	6
F130A	0.78 ± 0.01⁎	3	0.48 ± 0.03⁎	3
Y143A	0.20 ± 0.02	3	0.18 ± 0.04	3
E170A	0.11 ± 0.02	3	0.21 ± 0.03	3
I183A	0.16 ± 0.05	3	0.23 ± 0.12	3

⁎ Significantly different to WT, *p* <0.05; NB = no binding.

**Table 3:** 5-HT concentration-response parameters of 5-HT_3_A and 5-HT_3_AB receptors

Receptor	EC_50_ (μM)	Hill Coefficient	*n*
*5-HT3A subunit mutations*			
WT 5-HT_3_A	0.32 ± 0.05	2.3 ± 0.2	8
Y73A	0.088 ± 0.018⁎	2.1± 0.3	5
Y73F	0.45 ± 0.07	2.3 ± 0.3	5
Y73S	0.074 ± 0.016⁎	2.0 ± 0.3	3
W90C	NF		3
F130A	0.13 ± 0.03	1.5 ± 0.2	4
F130Y	NF		12
S163A	0.16 ± 0.03	3.8 ± 0.4	3
S163T	0.094 ± 0.026	2.5 ± 0.3	2
D165A	0.19 ± 0.03	1.8 ± 0.2	3
D165 K	0.19 ± 0.09	3.0 ± 0.3	6
W183C	NF		6

*5-HT3B subunit mutations*			
WT 5-HT_3_AB	0.50 ± 0.1	1.3 ± 0.2	11
H73A	0.64 ± 0.3	1.6 ± 0.2	7
W90C	0.13 ± 0.1	2.0 ± 0.4	4
F130A	2.4 ± 1.4	1.4 ± 0.2	5
Y143A	3.2 ± 1.7	1.3 ± 0.2	9
E170A	2.6 ± 1.7	1.4 ± 0.2	5
I183A	2.3 ± 0.4	1.2 ± 0.2	4

⁎ Significantly different to WT, *p* <0.05. NF = non functional. WT = wild type.

**Table 4:** Palonosetron concentration-inhibition parameters of 5-HT_3_A and 5-HT_3_AB receptors

Receptor	IC_50_ (nM)	*n*
*5-HT3A subunit mutations*		
WT 5-HT_3_A	0.83 ± 0.49	5
Y73A	13.1 ± 1.6⁎	3
Y73F	1.23 ± 0.15	4
Y73S	3.64 ± 1.1	3
W90C	NF	3
F130A	4.40 ± 1.8	3
F130Y	NF	9
S163A	1.57 ± 0.12	3
S163T	1.02 ± 0.19	3
D165A	1.33 ± 0.45	3
D165 K	1.30 ± 0.43	3
W183C	NF	3

*5-HT3B subunit mutations*		
WT 5-HT_3_AB	0.88 ± 0.15	11
H73A	0.97 ± 0.26	7
W90C	0.37 ± 0.10	4
F130A	0.84 ± 0.11	5
Y143A	0.38 ± 0.05	9
E170A	0.36 ± 0.11	5
I183A	0.34 ± 0.08	4

⁎ Significantly different to WT, *p* <0.05. NF = non functional. WT = wild type.

## References

[1] Lummis S.C. (2012). J. Biol. Chem..

[2] Thompson A.J., Lester H.A., Lummis S.C. (2010). Q. Rev. Biophys..

[3] Barnes N.M., Hales T.G., Lummis S.C., Peters J.A. (2009). Neuropharmacology.

[4] Thompson A.J., Lummis S.C. (2006). Curr. Pharm. Des..

[5] Thompson A.J., Lummis S.C. (2007). Expert Opin. Ther. Targets.

[6] Wong E.H., Clark R., Leung E., Loury D., Bonhaus D.W., Jakeman L., Parnes H., Whiting R.L., Eglen R.M. (1995). Br. J. Pharmacol..

[7] Rojas C., Stathis M., Thomas A.G., Massuda E.B., Alt J., Zhang J., Rubenstein E., Sebastiani S., Cantoreggi S., Snyder S.H., Slusher B. (2008). Anesth. Analg..

[8] Lummis S.C., Thompson A.J. (2013). Neuropharmacology.

[9] Hothersall J.D., Moffat C., Connolly C.N. (2013). Br. J. Pharmacol..

[10] Moura Barbosa A.J., De Rienzo F., Ramos M.J., Menziani M.C. (2010). Eur. J. Med. Chem..

[11] Price K.L., Lummis S.C. (2005). J. Neurosci. Methods.

[12] Brady C.A., Stanford I.M., Ali I., Lin L., Williams J.M., Dubin A.E., Hope A.G., Barnes N.M. (2001). Neuropharmacology.

[13] Thompson A.J., Price K.L., Lummis S.C. (2011). J. Physiol..

[14] Davies P.A., Pistis M., Hanna M.C., Peters J.A., Lambert J.J., Hales T.G., Kirkness E.F. (1999). Nature.

[15] Dubin A.E., Huvar R., D’Andrea M.R., Pyati J., Zhu J.Y., Joy K.C., Wilson S.J., Galindo J.E., Glass C.A., Luo L., Jackson M.R., Lovenberg T.W., Erlander M.G. (1999). J. Biol. Chem..

[16] Hapfelmeier G., Tredt C., Haseneder R., Zieglgansberger W., Eisensamer B., Rupprecht R., Rammes G. (2003). Biophys. J..

[17] Spier A.D., Lummis S.C. (2000). J. Biol. Chem..

[18] Rojas C., Thomas A.G., Alt J., Stathis M., Zhang J., Rubenstein E.B., Sebastiani S., Cantoreggi S., Slusher B.S. (2009). Eur. J. Pharmacol..

[19] Thompson A.J., Chau P.L., Chan S.L., Lummis S.C. (1979). Biophys. J..

[20] Price K.L., Lummis S.C. (2004). J. Biol. Chem..

[21] De Rienzo F., Barbosa A.J., Perez M.A., Fernandes P.A., Ramos M.J., Menziani M.C. (2012). J. Biomol. Struct. Dyn..

[22] De Rienzo F., Del Cadia M., Menziani M.C. (2012). Phys. Chem. Chem. Phys..

[23] Sullivan N.L., Thompson A.J., Price K.L., Lummis S.C. (2006). Mol. Membr. Biol..

[24] Steward L.J., Boess F.G., Steele J.A., Liu D., Wong N., Martin I.L. (2000). Mol. Pharmacol..

[25] Barrera N.P., Herbert P., Henderson R.M., Martin I.L., Edwardson J.M. (2005). Proc. Natl. Acad. Sci. U.S.A..

[26] Lochner M., Lummis S.C. (2010). Biophys. J..

[27] Miles T.F., Dougherty D.A., Lester H.A. (2013). Biophys. J..

[28] Thompson A.J., Verheij M.H., de Esch I.J., Lummis S.C. (2012). J. Pharmacol. Exp. Ther..

